# IUC26667-92 Assessment of QRISK®3 and Major Cardiovascular Events in Patients with Metastatic Hormone-Sensitive Prostate Cancer Treated with ADT ± ARPI ± Docetaxel: A Retrospective Cohort Study

**DOI:** 10.1093/oncolo/oyag286.014

**Published:** 2026-08-21

**Authors:** T Alom, A Kutty, G Neola, L Pedapati, A Sandulache, W The-Zar, C Chau, A Maniam, B Smalley, G L Banna

**Affiliations:** Portsmouth University Hospital NHS Trust - United Kingdom; Portsmouth University Hospital NHS Trust - United Kingdom; Portsmouth University Hospital NHS Trust - United Kingdom; Portsmouth University Hospital NHS Trust - United Kingdom; Portsmouth University Hospital NHS Trust - United Kingdom; Portsmouth University Hospital NHS Trust - United Kingdom; Portsmouth University Hospital NHS Trust - United Kingdom; Portsmouth University Hospital NHS Trust - United Kingdom; Portsmouth University Hospital NHS Trust - United Kingdom; Portsmouth University Hospital NHS Trust - United Kingdom

## Abstract

**Background:**

Cardiovascular disease is a major source of morbidity in patients with metastatic hormone-sensitive prostate cancer (mHSPC) receiving androgen deprivation therapy (ADT)-based treatment. Although cardiovascular risk assessment is increasingly recommended before treatment initiation, its integration into routine therapeutic decision-making remains limited. We evaluated baseline cardiovascular risk using QRISK®3 and its association with major adverse cardiovascular events (MACE) in patients receiving ADT-based treatment for mHSPC.

**Methods:**

We retrospectively evaluated 206 patients with mHSPC treated with ADT, with or without androgen receptor pathway inhibitors and/or docetaxel, at Portsmouth Hospitals University NHS Trust between 2009 and 2023. Demographic, clinical and cardiovascular variables were collected to calculate baseline QRISK®3. The primary endpoints were the distribution of baseline QRISK®3 categories and the incidence of MACE according to baseline cardiovascular risk. Secondary endpoints included the description of clinical characteristics across QRISK®3 categories, prescribing patterns of GnRH agonists versus antagonists according to cardiovascular risk, and the incidence of MACE within the first 36 months of ADT.

**Results:**

Among 206 patients, 113 (54.9%) were classified as high cardiovascular risk, 82 (39.8%) as intermediate risk and 11 (5.3%) as low risk. Patients with high QRISK®3 experienced a significantly higher incidence of MACE than those with intermediate/low QRISK®3 (17.7%vs6.5%; OR 3.12, 95%CI 1.20-8.14; p  =  0.020). This association remained significant in the prespecified 36-month landmark analysis (22.0% vs 6.8%; p  =  0.008), as well as in univariable (HR 2.94, 95%CI 1.16-7.14; p  =  0.022) and multivariable Cox regression analyses (adjusted HR 3.45, 95%CI 1.33-9.09; p  =  0.011). Among patients classified as high cardiovascular risk, only 45.1% were receiving statin therapy at baseline. Likewise, baseline cardiovascular risk did not appear to influence clinicians' choice between GnRH agonists and antagonists, with a comparable distribution of ADT agents across QRISK®3 categories (p  =  0.588).

**Conclusions:**

More than half of patients initiating ADT for mHSPC had a high baseline cardiovascular risk, which independently identified patients at increased risk of MACE. Nevertheless, cardiovascular risk appeared to have a limited influence on routine clinical management, as more than half of high-risk patients were not receiving statin therapy and did not appear to influence clinicians' choice between GnRH agonists and antagonists. These findings support routine cardiovascular risk stratification before ADT initiation.

